# Medical Items

**Published:** 1892-04

**Authors:** 


### *MEDICAL ITEMS.*

---

It is officially estimated that the influenza in New York State destroyed upwards of 10,000 victims in 1891.

---

Dr. G. Frank Lydston, Chicago, says that the only remedies that can be relied upon to check night sweats are Dover's powder and picrotoxin. Of these the former is more valuable.

---

The Journal received a pleasant call in March from Dr. J. C. LeGrand, of Anniston, the editor of our esteemed contemporary, the *Alabama Medical and Surgical Age.*

---

The Annals of Surgery, heretofore published "Simultaneously in St. Louis and London," has been sold to the University of Pennsylvania Press, and will henceforth be issued from Philadelphia.

---

It is said that the late Sir Morell Mackenzie received for his services to the Emperor Frederick the sum of $60,000. He afterwards received $500 for amputating the elongated uvula of an eminent lawyer.

---

Prof. Parvin advocates the use of the ordinary male catheter, instead of the female, as by its greater length the bladder can be emptied without soiling the clothes or necessitating any exposure of the patient.—*Ex.*

THE Thirty-ninth Annual Meeting of the Medical Society of the State of North Carolina will be held at Wilmington, on the 17th, 18th and 19th of May. Subject for debate, "Puerperal Eclampsia." Leader of debate, Frank W. Brown, M. D.

---

THE GRADY HOSPITAL is now completed, and was formally presented to the city April 1st. We had intended to publish in this issue of THE JOURNAL a detailed description of the hospital, but circumstances made it impossible to do so. It will appear next month.

---

Beginning with the Fall term of 1892, the four-year's course at the Harvard Medical School goes into effect. At this school during the session just past there were sixty-five applicants for the medical degree in the three-years course, of whom sixteen were rejected.

---

A man who breathes at the rate of 152 times per minute has been found in New York and presented by Dr. Janeway at the Bellevue clinic. The cause of this extraordinary breathing is supposed to be a lesion of the medulla oblongata resulting from an injury sustained about two years ago. The man describes himself as breathing "like a steam-engine under high pressure."

---

AS WE GO TO PRESS, we learn of the death of Dr. D. Hayes Agnew, of Philadelphia, who, at the time of his death, was Professor of Surgery in the University of Pennsylvania. The name Agnew is a deservedly honorable one in American medicine and surgery, and there has been no worthier representative of it than Dr. Hayes Agnew, of Philadelphia. In spite of a very large practice he found time to contribute a great deal to medical literature. His principal work was his *Treatise on Surgery*, in three volumes. The medical profession everywhere will regret the loss of Dr. Agnew.

---

### THE DOCTOR AND THE LAWYER.

[At the annual banquet closing the recent session of the Southern Medical College Mr. Charles A. Read, of the Atlanta bar, responded to the toast, "The Law Department," and appropriately finished his remarks with the following lines :]

When doctors meet to talk and eat
Around the social table,
'Tis surely due that lawyers, too,
Should join their charming Babel.

The doctor fills the human tills
With coin of bone and tissue;
And guarantees, with painless ease,
To bring forth every issue.

With patient care he watches near,
His visits oft diurnal,
Till ev'ry coin from father's loin
Is stamped in mint maternal.

Time flies apace; the father's race
Its earthly course is ending;
The doctor's skill cannot avail
To stay the stroke impending.

The lawyer then, with legal ken,
Doth make secure provision,
That every heir shall take his share
In papa's long division.

The mother, too, as is her due,
Is lawfully considered,
And takes, as wife, one-third for life,
If she by death be widowed.

The will is signed ; the father's mind
Is freed from cares terrestrial,
And soaring high beyond the sky
Doth enter fields celestial.

Be honor, then, by noble men,
To doctors, lawyers given;
Who first prepare for earthly care,
And then speed safe to heaven.

